# The Analysis of B-Cell Epitopes of Influenza Virus Hemagglutinin

**Published:** 2016

**Authors:** D.N. Shcherbinin, S.V. Alekseeva, M.M. Shmarov, Yu.A. Smirnov, B.S. Naroditskiy, A.L. Gintsburg

**Affiliations:** Federal State Budgetary Institution “Federal Research Centre for Epidemiology and Microbiology named after the honorary academician N.F. Gamaleya” of the Ministry of Health of the Russian Federation, Gamaleya str. 18, Moscow, Russian Federation, 123098

**Keywords:** Influenza virus, hemagglutinin, conformational epitopes, broad-spectrum monoclonal antibodies, broad-spectrum single-domain antibodies

## Abstract

Vaccination has been successfully used to prevent influenza for a long time. Influenza virus hemagglutinin (HA), which induces a humoral immune response in humans and protection against the flu, is the main antigenic component of modern influenza vaccines. However, new seasonal and pandemic influenza virus variants with altered structures of HA occasionally occur. This allows the pathogen to avoid neutralization with antibodies produced in response to previous vaccination. Development of a vaccine with the new variants of HA acting as antigens takes a long time. Therefore, during an epidemic, it is important to have passive immunization agents to prevent and treat influenza, which can be monoclonal or single-domain antibodies with universal specificity (broad-spectrum agents). We considered antibodies to conserved epitopes of influenza virus antigens as universal ones. In this paper, we tried to characterize the main B-cell epitopes of hemagglutinin and analyze our own and literature data on broadly neutralizing antibodies. We conducted a computer analysis of the best known conformational epitopes of influenza virus HAs using materials of different databases. The analysis showed that the core of the HA molecule, whose antibodies demonstrate pronounced heterosubtypic activity, can be used as a target for the search for and development of broad-spectrum antibodies to the influenza virus.

## INTRODUCTION

Hemagglutinin (HA) is the main antigenic component of influenza viruses. It is a homotrimeric mushroom- shaped surface glycoprotein whose monomer consists of two fragments linked by a disulfide bridge: HA1 (330 amino acids), the globular portion distal from the viral membrane, and HA2 (220 amino acids), the stem portion anchored in the viral membrane. Eighteen subtypes of influenza A virus HA were naturally found [1].

Virus-neutralizing antibodies induced by HA form the basis of humoral immunity, which protects the body against influenza infection [2]. The antigenic structure of HA is continuously changing as a result of the selective pressure of the immune system of the host organism, which leads to occurrence and selection of new variants of the virus capable of avoiding the neutralizing effect of available antibodies and overcoming the specific immune defense in humans. This mechanism, known as antigenic drift, reduces the effect of vaccination against influenza [3]. When pandemic strains of influenza A virus emerge and a virus with a new antigenic subtype of HA enters the human population (antigenic shift) [2, 5], the existing vaccines are ineffective. These factors explain the need for new approaches to the development of new broad-spectrum influenza drugs [5]. One of these approaches includes a search for and characterization of conserved antigenic determinants in the influenza virus HA molecule and development of broad-spectrum neutralizing antibodies. These antibodies can be used for emergency passive immunization and, therapy, when taking anti-epidemic measures.

Molecular studies of antigenic structures of HA have shown that the sites interacting with antibodies are mainly located in the globular domain of the HA1 subunit [6]. The amino acid sequences of these sites are extremely variable and differ not only in different HA subtypes, but also within the same subtype. Conserved determinants were found in the HA2 subunit [7-10]. These data suggest that conserved antigenic sites in the HA molecule can induce formation of antibodies with broad cross-neutralizing activity. This assumption was confirmed by Y. Okuno *et al*. [11], who first obtained and characterized a monoclonal antibody to the H2 subtype of HA, having neutralizing activity against influenza A virus strains with H2 and H1 HAs. This monoclonal antibody (C179) recognizes a conformational epitope in the stem region of the HA molecule, which is conserved in the H2 and H1 subtypes of influenza A viruses. It is known that the H5 and H6 subtypes of avian influenza viruses are phylogenetically close to the strains of the H1 and H2 subtypes [12, 13]. Broad-spectrum action of murine antibody S179 was identified at the D.I. Ivanovskiy Research Institute of Virology. It was shown that this antibody interacts with the H1, H2, and H5 subtypes (and even with the H6 subtype of HA in its not-fully-mature form) [14, 15].

Single-domain antibodies are considered to be promising agents for passive immunization against influenza. Single-domain antibodies are small, stable, and easy to produce. It was shown that intranasal administration of llama-derived single-stranded fragments of the variable domains of immunoglobulins, having a neutralizing activity *in vitro *against H5N1 influenza viruses, can control viral replication and reduce the incidence of the disease and mortality in mice infected with the H5N1 influenza virus. Although the study focused on single-domain antibodies recognizing the epitope near the receptor-binding domain, the possibility of selecting molecules of broad-spectrum antibodies that bind to other HA epitopes, including conserved ones, was emphasized [16].

In another study, a single-domain antibody to influenza A virus HA was produced and a recombinant adenovirus expressing this antibody was designed. Administration of this recombinant adenovirus in the period 48 hours to 14 days before the challenge can fully protect mice against influenza A virus [17].

Therefore, there is an obvious need for the search for passive immunization agents with universal specificity, which could allow us to circumvent the antigenic variability of the influenza virus [18].

It is possible that the direction of the search for ways of passive immunization providing protection against a broad spectrum of influenza viruses, which is being conducted in cooperation between Japanese and Russian scientists and is currently underway in several laboratories, will turn out to be the most promising.

## ANTIBODIES TO THE GLOBULAR PORTION OF HA MOLECULE

**Table 1:** Known antibodies to B-cell epitopes of influenza A and B virus HAs

Antigenic site	Antibody	PDB ID	Source of antibodies	Antibody subtype	Source of antigen	Reference
R.b.p.	CH65, CH67	3SM5	H. s.	IgG1	A/Solomon Islands/3/2006(H1N1)	[27,33]
R.b.p.	CH65	3SM5	H. s.	IgG1	A/Solomon Islands/3/2006(H1N1)	[27]
R.b.p.	CH67	4HKX	H. s.	IgG1	A/Solomon Islands/3/2006(H1N1)	[33]
Sasite	2D1	3LZF	H. s.	?	A/South Carolina/1/1918(H1N1)	[19]
R.b.p.	1F1	4GXU	H. s.	?	A/South Carolina/1/1918(H1N1)	[28]
	GC0757	4F15	M. m.	?	A/California/04/2009(H1N1)	[45]
	GC0587	4LVH	M. m.	?	A/California/04/2009(H1N1)	[23]
R.b.p.	5J8	4M5Z	H. s.	?	A/California/07/2009(H1N1)	[26]
R.e.s.	H5M9	4MHH	M. m.	IgG1	A/Viet Nam/1203/2004 (H5N1)	[20]
	H5M9	4MHJ			A/goose/Guangdong/1/1996(H5N1)	[20]
R.b.p.	8F8	4HF5	H. s.	?	A/Japan/305+/1957(H2N2)	[29]
R.b.p.	8M2	4HFU	H. s.	?	A/Japan/305+/1957(H2N2)	[29]
R.b.p.	2G1	4HG4	H. s.	?	A/Japan/305+/1957(H2N2)	[29]
R.e.s.	BH151	1EO8	M. m.	IgG1	A/X-31(H3N2)	[22]
R.e.s.	HC45	1QFU	M. m.	IgG1	A/X-31(H3N2)	[21]
R.b.p.	C05	4FP8 4FQR	H. s.	?	A/Hong Kong/1/1968(H3N2)	[30]
R.b.p.	S139/1	4GMS	M. m.	IgG2a	A/Victoria/3/1975(H3N2)	[31]
R.b.p.	F045-092	4O58	H. s.	?	A/Victoria/3/1975(H3N2)	[32]
R.b.p.	F045-092	4O5I	H. s.	?	A/Singapore/H2011.447/2011(H3N2)	[32]
R.b.p.	HC63	1KEN	M. m.	?	A/X-31(H3N2)	[46]
R.b.p.	HC19	2VIR 2VIS 2VIT	M. m.	IgG1	A/X-31(H3N2)	[47]
	IIB4		M. m.	?	A/Philippines/2/1982(H3N2)	[48]
	Fab 26/9	1FRG	M. m.	IgG2a	A/Victoria/3/1975(H3N2)	[49]
	CR8071	4FQJ	H. s.	IgG1	B/Florida/4/2006	[44]
	CR8059	4FQK	H. s.	IgG1	B/Brisbane/60/2008	[44]
S.p.	FI6v3	3ZTJ	H. s.	?	A/Aichi/2/1968(H3N2)	[42]
	FI6v3	3ZTN	H. s.	?	A/California/04/2009(H1N1)	[42]
	MAb 3.1	4PY8	H. s.	IgG1	A/South Carolina/1/1918(H1N1)	[39]
	CR6261	3GBN	M. m.	IgG1	A/Brevig Mission/1/1918(H1N1)	[37, 50]
	CR6261	3GBM	M. m.	IgG1	A/Viet Nam/1203/2004(H5N1)	[37]
	CR8020	3SDY	H. s.	?	A/Hong Kong/1/1968(H3N2)	[40]
	F10	3FKU	H. s.	IgG1	A/Viet Nam/1203/2004(H5N1)	[38]
	C179	4HLZ	M. m.	IgG2a	A/Japan/305/1957(H2N2)	[11, 15, 36]
	CR8043	4NM8	H. s.	IgG1	A/Hong Kong/1/1968(H3N2)	[41]
	CR9114	4FQI	H. s.	IgG1	A/Viet Nam/1203/2004(H5N1)	[44]
	CR9114	4FQV	-//-//-	-//-//-	A/Netherlands/219/2003(H7N7)	[44]
	CR9114	4FQY	-//-//-	-//-//-	A/Hong Kong/1/1968(H3N2)	[44]
	Fab 39.29	4KVN	H. s.	?	A/Perth/16/2009(H3N2)	[43]

Information on the best known conformational B-cell epitopes of influenza virus HAs was obtained from the database of immunological epitopes (IEDB – Immune Epitope Database and analysis resource www.iedb.org) and the protein database (PDB – Protein Data Bank; www.rcsb.org). The H1 subgroup of influenza viruses (H1, H5, and H2) is colored in blue, the H3 subgroup is colored in green; and antibodies to the stem portion of HAs of different influenza viruses are colored in red. S.p. – stem portion. R.b.p. – receptor binding pocket, R.e.s. – the site localized in a rudimentary esterase subdomain, H. s. – Homo sapiens, M. m. – Mus musculus.

The largest amount of virus-neutralizing antibodies produced in a natural or artificial way bind to the globular portion of HA, resulting in blockage of virion binding to cells. However, since the *HA *gene rapidly mutates, new amino acid substitutions occur, leading to formation of new glycosylation sites, which in turn causes changes in the surface structure of the protein. Therefore, these antigenic sites are highly variable and the corresponding antibodies are strain-specific. This partially explains why immunity after natural infection or vaccination is largely limited to the circulating strain. For example, 2D1 antibodies binding to the Sa-antigenic site located in the globular portion of the HA molecule recognize only the pandemic H1N1 viruses of 1918 and 2009, whose epitopes are antigenically similar, although they are separated by almost a century [19]. Other H1 strains, such as PR8, cannot be recognized by these antibodies *(Table 1)*, *(Figure)*.

**Fig. 1:**
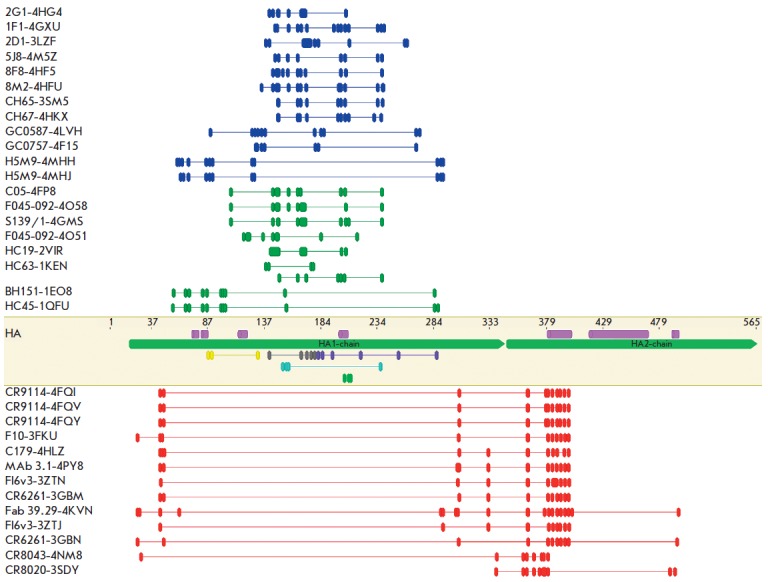
Schematic arrangement of B-cell epitopes in the amino acid sequence of hemagglutinin (HA). The figure was obtained using the Geneious 9.0.2 software as follows: the amino acid sequences of influenza virus hemagglutinins recognized by the corresponding antibodies (Table 1) were aligned with respect to each other. The epitope recognized by the corresponding antibody was mapped on each sequence. Information on the B-cell epitopes of HAs was obtained from the immunological epitope database. The figure in the middle shows HA with the following elements: HA1 and HA2 chains (green), alpha helices (pink), antigenic sites: Cb (yellow), Ca1 (purple), Ca2 (blue), Sa (gray), and Sb (green). Location of B-cell epitopes of influenza A virus HA is shown above and below the HA (see Table 1). H1 (dark blue) and H3 (dark green) epitopes to the globular portion of HA are shown above; epitopes to the stem portion of HA (dark red) are shown below. Antigenic sites are mapped according to Caton A.J. [50].

However, several antibodies specific to the globular part of HA and having virus-neutralizing activity against several strains of the influenza virus within one subtype have been recently described and characterized. These epitopes are conserved between different viral strains and, therefore, are recognized by the same antibody. It is noteworthy that the epitopes of these antibodies can be located at different antigenic sites. For example, H5M9 antibodies interact with the conserved H5 subtype of the HA epitope, which is located in the rudimentary esterase subdomain in close vicinity to the receptor-binding site and partially overlaps the antigenic site Cb [20]. H5M9 antibodies effectively protect mice against lethal doses of different H5 strains. HC45 and BH151 antibodies also interact with similar antigenic sites *(Figure)*, but their ability to interact with different strains was not identified [21, 22]. GC0757 and GC0857 antibodies interact with the same epitope located in the globular portion of HA and recognize various H1 strains [23]. These antibodies interact with a previously unknown epitope which is not located in the known antigenic sites [23].

A number of other antibodies interacting with several strains of the influenza virus recognize the receptor- binding site in the globular part of HA. Since the receptor-binding site is functionally conserved, its amino acid diversity is limited and it is regarded as an attractive target for broad-spectrum antibodies [24].

The receptor-binding site is a wide, shallow pocket localized at the top of the globular domain. The boundaries of the receptor binding site are formed by the loops 130, 150, and 220 and α-helix 190, which indicate positions in the amino acid sequence of HA [25]. Structural characterization of some antibodies bound to the receptor-binding site showed that all antibodies build a variable loop into the receptor-binding site and, thus, directly block the interaction of HA with cellular sialic acids [26-32]. However, most antibodies interact with only one loop due to the compact structure of the site, and only few antibodies interact with two loops. Since the receptor-binding site is located in the globular part of HA, it creates no steric barrier to the formation of antibodies to this antigenic site.

1F1 antibodies were obtained from people who had influenza during the pandemic in 1918. These antibodies can inhibit some strains of the H1 influenza A virus; namely those isolated in 1918, 1943, 1947, and 1977 [28]. The study of the crystal structure of these antibodies in a complex with influenza virus HA from 1918 has shown that they interact with amino acid residues that belong to the antigenic sites Sa, Sb, and Ca2. The heavy chain of the 1F1 antibody also comes into contact with the receptor-binding site and interacts with the amino acid residues involved in the binding to sialic acids.

The CH65 and CH67 antibodies bind and neutralize H1 influenza viruses, which have circulated in the human population since 1986 [27, 33]. However, these antibodies are not active against the 2009 H1pandemic influenza virus. The 5J8 antibodies are active against the H1 subtype of HAs of both the 1918 and 2009 pandemic influenza viruses and seasonal influenza A viruses. The study of the crystal structure of the CH65, CH67, and 5J8 antibodies in a complex with HA revealed that they recognize epitopes near the receptor-binding site and build their HCDR3 loop into the receptor-binding pocket.

H2N2 viruses circulated in the human population for 11 years, from 1957 to 1968. Since these viruses remained absent for a long time, population immunity decreased significantly. Moreover, it is completely absent in people born after 1968: so, the probability of a reappearance of this subtype of the virus is very high, which undoubtedly raises concern. Antibodies to the H2N2-8F8, 8M2, and 2G1 subtypes, which recognize and neutralize all subtypes of H2 HA from 1957 to 1968, were obtained from donors using hybridoma technology [29]. The analysis of the crystal structures of these antibodies in a complex with HA showed that they recognize the receptor-binding pocket. Antibodies to 8F8 insert their HCDR3 loop into the receptor-binding pocket, whereas antibodies 8M2 and 2G1 insert their HCDR2 loop [29]

The aforementioned antibodies to the receptor-binding pocket of HA indicate that this portion of the globular part of HA is more conserved than the antigenic sites Sa or Sb, but that antibodies to this site are unable to recognize different HA subtypes. Nevertheless, antibodies that recognize the receptor-binding pocket and are capable of heterosubtypic recognition of HA were found.

C05 and S139/1 antibodies have heterosubtypic activity and can bind to many influenza virus subtypes, including H1, H2, and H3 [30, 31]. S139/1 antibodies were obtained from mice immunized with the H3N2 virus. These are the first heterosubtypic antibodies that recognize the receptor-binding pocket by interacting with the H1, H2, H3, H5, and H13 subtypes of HA [34]. The analysis of the crystal structures of these antibodies in a complex with HA has shown that they interact with the receptor-binding pocket through the HCDR2 loop [31]. The study of the binding and neutralization of the virus confirmed that S139/1 antibodies do have heterosubtypic activity, although with narrow specificity within one subtype. Nevertheless, these results suggest that different strains of different subtypes of the influenza A virus may contain a similar epitope within the receptor-binding site.

Other antibodies, C05, were found using a phage library prepared based on cells isolated from individuals infected with the seasonal influenza virus [30]. C05 have neutralizing activity against the H1, H2, H3, and H9 viruses and have a greater breadth of recognition within these subtypes compared to S139/1 antibodies. Unlike other previously described antibodies recognizing the receptor-binding pocket, C05 bind to HA exclusively through the heavy chain. The main interaction is mediated only by a long HCDR3 loop, which penetrates into the receptor-binding pocket. The epitope for these antibodies on the HA surface is very compact.

Still another group of antibodies with broad heterosubtypic recognition, F045-092, were also obtained using phage libraries based on cells isolated from donors. They are able to recognize and neutralize various strains of the H1, H2, H3, and H5 subtypes of the influenza virus [35]. Analysis of the crystal structure of F045-092 antibodies in a complex with HA showed that they insert the HCDR3 loop into the receptor-binding pocket, wherein the carboxyl group of an aspartate at the apex of the recognition loop mimics the carboxyl group of the sialic acid [32]. Broader recognition of different subtypes of the influenza A virus is probably achieved due to the receptor mimicry.

## ANTIBODIES TO THE STEM PART OF THE HA MOLECULE

The originally described antigenic sites were located only in the globular domain of HA, and the viewpoint that the stem region is not accessible for a humoral immune response was wide spread. However, in 1993, S179 antibodies were described which were obtained from mice immunized with the H2N2 influenza virus and were capable of neutralizing the H1, H2, and H5 subtypes of HA [11, 14]. In contrast to the antibodies to the globular part, these antibodies blocked the conformational reorganization of HA at a low pH, thus inhibiting its function. Twenty years after the discovery of the S179 antibodies, their crystal structure in a complex with the H5 subtype of HA was determined. The analysis of this complex showed that the antibodies interact with HA using both heavy and light chains [36].

Fifteen years after the discovery of the S179 of antibodies, several additional antibodies to the stem portion of HA were described. Investigation of the structure of two of these human antibodies, CR6261 [37] and F10 [38], in a complex with HA showed that they interact with a highly conserved epitope in the stem portion, which is similar in all first-group HAs *(Table2). *Both antibodies interact with HA only through the heavy chain, inserting the HCDR2 loop into the hydrophobic pocket.

**Table 2:** Interaction between monoclonal antibodies specific to the stem portion of HA and different subtypes of influenza A viruses.

Group classification of influenza viruses		Monoclonal antibody								
		C179	F10	CR6261	MAb 3.1	CR8020	CR8043	Fab 39.29	FI6v3*	CR9114**
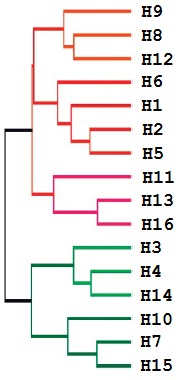	H9	n.t.	+	+	-	-	-	n.t.	n.t.	+
	H8	n.t.	+	+	-	-	-	n.t.	n.t.	+
	H12	-	n.t.	-	-	-	-	n.t.	n.t.	+
	H6	+	+	+	+	-	-	n.t.	n.t.	+
	H1	+	+	+	+	-	-	+	+	+
	H2	+	+	+	+	-	-	+	n.t.	+
	H5	+	+	+	+	-	-	+	+	+
	H11	n.t.	+	-	-	-	-	n.t.	n.t.	n.t.
	H13	-	-	-	-	-	-	n.t.	n.t.	n.t.
	H16	-	-	-	-	-	-	n.t.	n.t.	n.t.
	H3	-	-	-	-	+	n.t.	+	+	+
	H4	-	-	-	-	+	n.t.	n.t.	n.t.	+
	H14	-	-	-	-	n.t.	+	n.t.	n.t.	n.t.
	H10	-	-	-	-	+	n.t.	n.t.	n.t.	+
	H7	-	-	-	-	+	+	+	+	+
	H15	-	-	-	-	n.t.	+	n.t.	n.t.	n.t.

Note. “+”- Neutralization of influenza virus, “-” interaction with HA was not detected, “n.t.”– not tested. Group H1 influenza viruses are shown in red, group H3 antibodies are shown in green.

* FI6v3 antibodies interact with other subtypes of HA, but neutralization of the virus has not been investigated.

** CR9114 interacts with the influenza B virus, but it does not neutralize it.

Other monoclonal heterosubtypic antibodies (MAb 3.1) were obtained from donors using phage library. MAb 3.1 antibodies are able to neutralize H1a influenza viruses (H1, H2, H5, and H6), but they show weak neutralizing activity against the H1b subgroup (H13, H16, and H11) [39]. Similarly to other heterosubtypic antiinfluenza antibodies, CR6261 and F10, MAb 3.1 enters into contact with the stem part of HA, using only the heavy chain. In contrast, the HCDR1 and HCDR3 loops are involved in this interaction in MAb 3.1.

Antibodies that interact exclusively with the second group of HA were also discovered. For example, CR8020 antibodies, isolated from a healthy donor, bonded to the highly conserved epitope at the stem portion of HA and exhibited neutralizing activity against the H3, H7, and H10 viruses [39]. Later on, other antibodies were obtained: namely, CR8043, which, unlike CR8020, is encoded by other gene segments [40]. In experiments *in vitro, CR8043 *demonstrated neutralizing activity against the H3 and H10 subtypes of the influenza virus and protected mice against lethal doses of the H3N2 and H7N7 viruses [41]. The CR8020 and CR8043 antibodies bind to similar epitopes, but they interact with HA in different ways. Both antibodies interact with both the light and heavy chains of HA. Similarly to antibodies binding to the first group of HA, CR8020 and CR8043 antibodies also interact with the stem portion of the HA molecule and prevent conformational changes in it at a low pH. These antibodies may also inhibit the HA maturation process, blocking the proteolytic cleavage of immature precursor HA0 into the HA1 and HA2 subunits. Therefore, epitopes of these antibodies, which have been found and structurally characterized, are the second critical area in the stem portion of the HA molecule.

Monoclonal antibodies having heterosubtypic activity against both the first (H1) and second (H3) groups of the influenza A virus were described. Such broadspectrum heterosubtypic antibodies, FI6v3, were first characterized in 2011. They were isolated from a library consisting of 104,000 plasma cells derived from eight donors using the single-cell culturing method [42]. FI6v3 antibodies show neutralizing activity against both groups of viruses and inhibit the formation of syncytium in a cell culture. Similar Fab-fragments of the monoclonal antibodies Fab 39.29 were obtained using “*in vitro *activation and antigen-specific enrichment” of 840 plasma blasts of vaccinated individuals [43]. ;

Yet other broad-spectrum heterosubtypic antibodies, CR9114, bind to the conserved epitope in the stem portion of HA and demonstrate activity against all tested influenza A virus strains in neutralization tests [44]. Moreover, these antibodies are capable of interacting with the influenza B virus. However, in experiments* in vitro, *neutralization of the influenza B virus was not detected, at least in the tested concentrations. Therefore, at present, CR9114 are antibodies with the broadest specificity among all known monoclonal antibodies to influenza A virus HA.

Heterosubtypic antibodies to the influenza B virus were also found. In particular, the CR8059 and CR8071 antibodies can neutralize influenza B viruses in both lines [44].

The possibility of obtaining single-domain antibodies with neutralizing cross-activity was first shown with respect to the H1, H2, H5, and H9 influenza virus subtypes. Four cross-neutralizing antibodies (R2b-E8, R2b-D9, and R1a-B6) were bound to the full-length HA, rather than the HA1 domain, and unbound at low pH. These antibodies can bind to epitopes in the membrane proximal region of the HA stem far from the receptor-binding site. This cross-neutralization mechanism was described for the human monoclonal antibodies F10 and CR6261. One of these antibodies (R2a- G8) binds to a portion of the HA1 domain located in the stem part of HA [18].

Based on the aforementioned data, all antibodies can be classified into four groups according to their breadth of recognition.

1) Antibodies to the globular portion recognizing one or a few strains within one HA subtype (2D1);

2) Antibodies to the globular portion recognizing a large number of strains or all strains within one HA subtype (H5M9, HC45, BH151,8F8, 8M2, 2G1, etc.);

3) Antibodies to the globular portion capable of recognizing several strains of different HA subtypes (C05 and S139/1); and

4) Antibodies to the stem portion reaching pronounced heterosubtypic activity (C179, F10, CR6261, CR8020, FI6v3, MAb 3.1, CR8043, Fab 39.29, and CR9114).

## CONCLUSION

The epidemic outbreaks of influenza that occasionally occur in vaccinated populations certainly demonstrate the need for continued search for agents for emergency prevention and treatment of this disease. In this regard, protectors against pandemic influenza strains are of particular importance.

The idea of the development of broad-spectrum agents that can neutralize various subtypes of influenza viruses is the most promising for emergency prevention of influenza caused by the virus, which is volatile against the major antigen hemagglutinin.

In this investigation, we studied the ability of neutralizing broad-spectrum antibodies to recognize various B-cell epitopes of HA, which is very important in view of the evolution of influenza viruses.

The computer analysis of known conformational Bcell epitopes of influenza virus HA has shown that the stem part of the HA molecule, whose antibodies have pronounced heterosubtypic activity, should be the target for the search for and development of broadspectrum antibodies to the influenza virus. CR9114 antibodies demonstrate the widest cross-neutralizing activity against influenza A virus HA, compared to all the monoclonal antibodies that are currently available and being investigated. The heterosubtypic antibodies CR8059 and CR8071 influenza B virus type have also been found.

These data indicate that it is possible to design broad-spectrum drugs for emergency prevention and treatment of influenza using monoclonal or single-domain antibodies neutralizing certain B-cell epitopes in the stem portion of influenza virus HA.

## Abbreviations

HA: influenza virus hemagglutinin
H1–H18: subtypes of influenza virus hemagglutinin
